# Supplementary material for: Regulation of Oncogenic Targets by miR-99a-3p (Passenger Strand of miR-99a-Duplex) in Head and Neck Squamous Cell Carcinoma
Source: Cells. 2019 Nov 28;8(12):1535. doi: 10.3390/cells8121535 (PMC6953126; doi:10.3390/cells8121535)
Supplement: Supplementary file 1 [file cells-08-01535-s001.pdf]

Figure S1

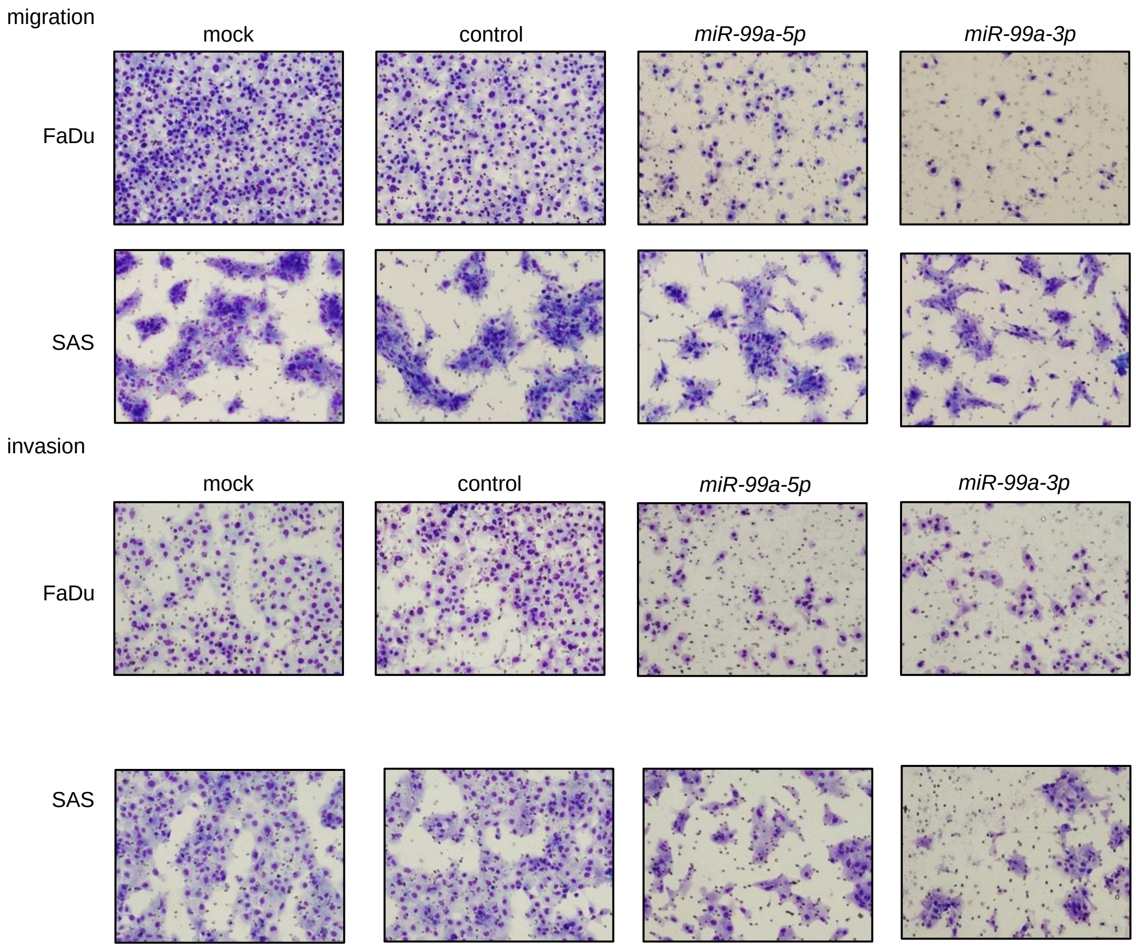

migration
mock
control
miR-99a-5p
miR-99a-3p
FaDu
SAS
invasion
mock
control
miR-99a-5p
miR-99a-3p
FaDu
SAS

Figure S1

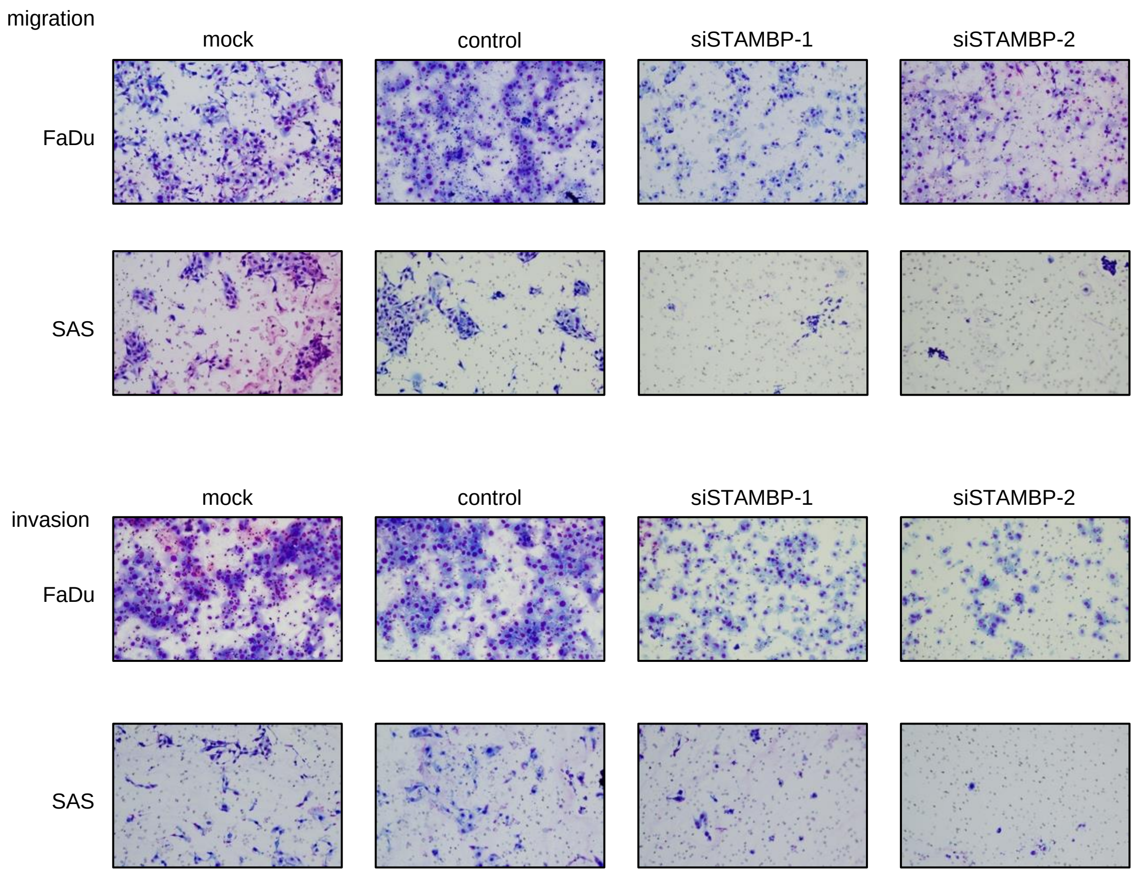
migration
mock
control
siSTAMBP-1
siSTAMBP-2
FaDu
SAS
invasion
mock
control
siSTAMBP-1
siSTAMBP-2
FaDu
SAS

Figure S2

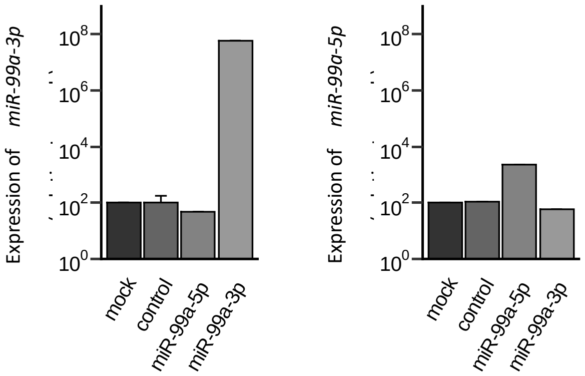

Expression of miR-99a-3p
10^8
10^6
10^4
10^2
10^0
mock
control
miR-99a-5p
miR-99a-3p
Expression of miR-99a-5p
10^8
10^6
10^4
10^2
10^0
mock
control
miR-99a-5p
miR-99a-3p

Figure S3

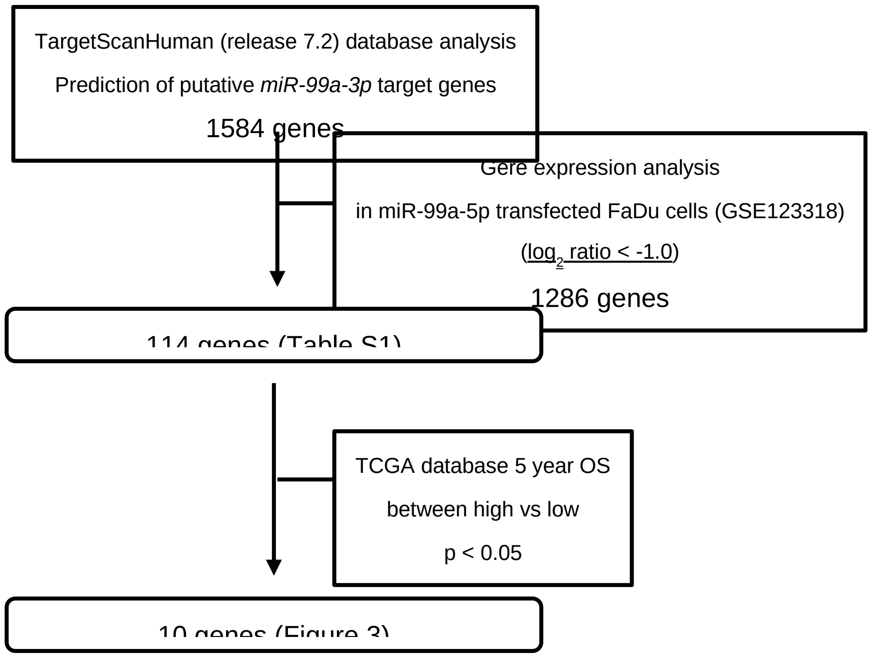

TargetScanHuman (release 7.2) database analysis
Prediction of putative *miR-99a-3p* target genes
1584 genes
Gene expression analysis
in miR-99a-5p transfected FaDu cells (GSE123318)
(log_2 ratio < -1.0)
1286 genes
114 genes (Table S1)
TCGA database 5 year OS
between high vs low
p < 0.05
10 genes (Figure 3)

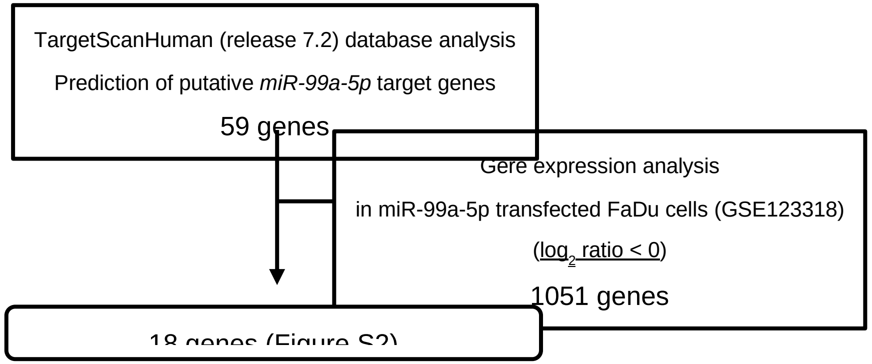

TargetScanHuman (release 7.2) database analysis
Prediction of putative *miR-99a-5p* target genes
59 genes
Gene expression analysis
in miR-99a-5p transfected FaDu cells (GSE123318)
(log_2 ratio < 0)
1051 genes
18 genes (Figure S2)

Figure S4

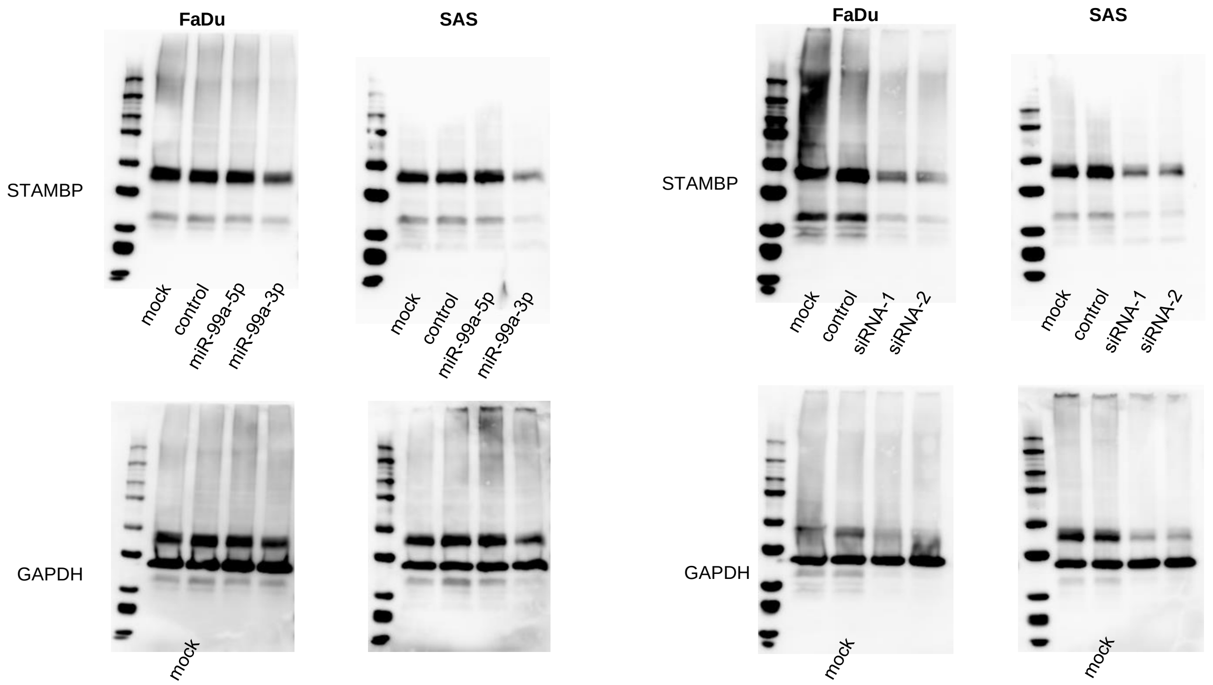
FaDu
SAS
FaDu
SAS
STAMBP
STAMBP
mock
control
miR-99a-5p
miR-99a-3p
mock
control
miR-99a-5p
miR-99a-3p
mock
control
siRNA-1
siRNA-2
mock
control
siRNA-1
siRNA-2
GAPDH
GAPDH
mock
mock
mock
mock

Figure S5

Inserted sequence

Wild type	GGTTTATGGCAATATGAATGGAGCTTATTACTGGGGTGAGGGACAGCGTTT
Deletion type	TTTCCTGTGGTTTATGGCA____________________TTACTGGGGTGAGGGACAGCTTACTCCAGTTT

Table 1. Candidate target genes regulated by *miR-99a-5p*.

Entrez Gene ID	GeneSymbol	GeneName	Total sites	GSE6631 FC (log2)	FaDu miR-99a-5p transfectant FC (log2)	TCGA OncoLnc 5-years OS p-value
23135	*KDM6B*	lysine (K)-specific demethylase 6B	1	-0.17771327	-0.594301	0.0720
2117	*ETV3*	ets variant 3	1	-0.01524691	-0.309083	0.1060
6304	*SATB1*	SATB homeobox 1	1	-0.4243871	-0.108264	0.1222
29841	*GRHL1*	grainyhead-like 1 (Drosophila)	1	#N/A	-0.153416	0.1238
64793	*CEP85*	centrosomal protein 85kDa	1	#N/A	-0.248518	0.1471
4154	*MBNL1*	muscleblind-like splicing regulator 1	1	-0.08976501	-0.101107	0.1513
11176	*BAZ2A*	bromodomain adjacent to zinc finger domain, 2A	2	-0.10055287	-0.418264	0.2698
27090	*ST6GALNAC4*	ST6 (alpha-N-acetyl-neuraminyl-2,3-beta-galactosyl-1,3)-N-acetylgalactosaminide alpha-2,6-sialyltransferase 4	1	0.12752819	-0.405108	0.2777
8467	*SMARCA5*	SWI/SNF related, matrix associated, actin dependent regulator of chromatin, subfamily a, member 5	1	0.0081548	-0.135667	0.7293
2475	*MTOR*	mechanistic target of rapamycin (serine/threonine kinase)	1	-0.03060691	-0.171068	0.7568
65084	*TMEM135*	transmembrane protein 135	1	0.23401306	-0.11437	0.7593

8897	*MTMR3*	myotubularin related protein 3	1	-0.28117563	-0.120421	0.7647
6602	*SMARCD1*	SWI/SNF related, matrix associated, actin dependent regulator of chromatin, subfamily d, member 1	1	0.1141998	-0.256544	0.7737
2289	*FKBP5*	FK506 binding protein 5	1	0.0416141	-0.257657	0.8489
23140	*ZZEF1*	zinc finger, ZZ-type with EF-hand domain 1	1	-0.11393538	-0.22949	0.078*
51029	*DESI2*	desumoylating isopeptidase 2	1	0.32899525	-0.171493	0.0289*
10482	*NXF1*	nuclear RNA export factor 1	1	-0.14998555	-0.273346	0.0126*
23507	*LRRC8B*	leucine rich repeat containing 8 family, member B	1	-0.05650079	-0.327775	0.0101*

*poor prognosis in patients with low gene expression

Table 2. Reagents used in this study.

**TaqMan primers and probes**	Assay ID		Company
hsa-miR-99a-5p	000435		Applied Biosystems, Waltham, Massachusetts, USA
hsa-miR-99a-3p	002141		Applied Biosystems, Waltham, Massachusetts, USA
RNU48	001006		Applied Biosystems, Waltham, Massachusetts, USA
STAMBP	Hs00197726_m1		Applied Biosystems, Waltham, Massachusetts, USA
GUSB	Hs99999908_m1		Applied Biosystems, Waltham, Massachusetts, USA
GAPDH	Hs02786624_g1		Applied Biosystems, Waltham, Massachusetts, USA
pre-miR miRNA Precursors	Assay ID	Concentration	
miR-99a-5p	PM10719	10nM	Thermo Fisher Scientific, Waltham, Massachusetts, USA
miR-99a-3p	PM12983	10nM	Thermo Fisher Scientific, Waltham, Massachusetts, USA
negative control miRNA #2	AM17111	10nM	Thermo Fisher Scientific, Waltham, Massachusetts, USA
Stealth RNAi siRNA	Assay ID	Concentration	
STAMBP	HSS116365<br>HSS116367	10nM	Invitrogen, Waltham, MA, USA
antibody	catalog number	dilution	
Anti-STAMBP	HPA035800	WB 1:750<br>IHC 1:500	Sigma-Aldrich, St. Louis, Missouri, USA
GAPDH	ab8245	WB 1:10000	Abcam, Cambridge, UK
